# A Self-Reported Screening Tool for Detecting Community-Dwelling Older Persons with Frailty Syndrome in the Absence of Mobility Disability: The FiND Questionnaire

**DOI:** 10.1371/journal.pone.0101745

**Published:** 2014-07-07

**Authors:** Matteo Cesari, Laurent Demougeot, Henri Boccalon, Sophie Guyonnet, Gabor Abellan Van Kan, Bruno Vellas, Sandrine Andrieu

**Affiliations:** 1 Institut du Vieillissement, Gérontopôle, Centre Hospitalier Universitaire de Toulouse, Toulouse, France; 2 Inserm UMR1027, Université de Toulouse III Paul Sabatier, Toulouse, France; Cardiff University, United Kingdom

## Abstract

**Background:**

The “frailty syndrome” (a geriatric multidimensional condition characterized by decreased reserve and diminished resistance to stressors) represents a promising target of preventive interventions against disability in elders. Available screening tools for the identification of frailty in the absence of disability present major limitations. In particular, they have to be administered by a trained assessor, require special equipment, and/or do not discriminate between frail and disabled individuals. Aim of this study is to verify the agreement of a novel self-reported questionnaire (the “Frail Non-Disabled” [FiND] instrument) designed for detecting non-mobility disabled frail older persons with results from reference tools.

**Methodology/Principal Findings:**

Data are from 45 community-dwelling individuals aged ≥60 years. Participants were asked to complete the FiND questionnaire separately exploring the frailty and disability domains. Then, a blinded assessor objectively measured the frailty status (using the phenotype proposed by Fried and colleagues) and mobility disability (using the 400-meter walk test). Cohen's kappa coefficients were calculated to determine the agreement between the FiND questionnaire with the reference instruments. Mean age of participants (women 62.2%) was 72.5 (standard deviation 8.2) years. Seven (15.6%) participants presented mobility disability as being unable to complete the 400-meter walk test. According to the frailty phenotype criteria, 25 (55.6%) participants were pre-frail or frail, and 13 (28.9%) were robust. Overall, a substantial agreement of the instrument with the reference tools (kappa = 0.748, quadratic weighted kappa = 0.836, both p values<0.001) was reported with only 7 (15.6%) participants incorrectly categorized. The agreement between results of the FiND disability domain and the 400-meter walk test was excellent (kappa = 0.920, p<0.001).

**Conclusions/Significance:**

The FiND questionnaire presents a very good capacity to correctly identify frail older persons without mobility disability living in the community. This screening tool may represent an opportunity for diffusing awareness about frailty and disability and supporting specific preventive campaigns.

## Introduction

The aging of our societies combined with the high costs of healthcare directed towards older persons (especially if disabled) represent major threats for the sustainability of public health services. In these last years, early actions aimed at implementing strategies against the onset of disabling conditions have been repeatedly advocated [1], [2]. Programs of primary prevention aimed at avoiding the beginning of the irreversible disabling process are indeed urgently needed. Obviously, they should target individuals who are not already experiencing the outcome of interest (i.e. disability), but are still exposed to specific risk factors for it. In this context, the so-called “frailty syndrome” is largely recognized as an interesting and promising pre-disability state to consider [3]. Frailty is described as a multi-systemic disruption of the organism's homeostasis and characterized by an extreme vulnerability to endogenous and exogenous stressors [4]. The detection of frail older individuals living in the community has repeatedly been advocated as the first step for building up effective prevention strategies against its negative health-related consequences (including falls, disability, institutionalizations, and mortality) [5], [6].

A wide range of instruments has been developed over the years for identifying frailty in the elderly. Unfortunately, available screening tools for the identification of non-disabled frail older persons still present two major limitations: 1) very few are valid for self-completion, and 2) none enables to differentiate frailty from disability.

As mentioned, although some exceptions exist (e.g., the PRISMA-7 tool [7], [8], or the Sherbrooke Postal Questionnaire [9]), most of the available instruments are not designed to be self-administered. This represents a relevant issue if the screening has to target large populations, such as in the case of preventive campaigns against disability requiring the evaluation of community-dwelling older persons. For example, the well-known frailty phenotype [10] is not feasible without the help of an assessor trained at 1) conducting the interview (including a complex questionnaire such as the Minnesota Leisure Time Activity for estimating the kcal/week of energy consumption [11]), and 2) administer the physical function tests (i.e. usual gait speed test and handgrip strength measurement). It is noteworthy that the frailty phenotype also requires the use of a dynamometer, which tends to be rarely available even in the clinical setting (especially in primary care). For facilitating the clinical implementation of the frailty syndrome, Ensrud and colleagues [12] proposed and validated the use of the three defining criteria (i.e. fatigue, involuntary weight loss, and chair stand test) in the Study of Osteoporotic Fractures (SOF). Nevertheless, such simplification did not still completely solve the problem concerning the need of an assessor/supervisor. In fact, there might be safety issues (e.g. risk of falls) at promoting the self-assessment of the chair stand test (i.e. ability to rise from a chair 5 times without using the arms) without supervision, especially if the target population may include frail older persons. The Gérontopôle Frailty Screening Tool (GFST) [13] and the 7-point Clinical Frailty Scale [14] again require the presence of an assessor. In particular, they are largely based on the subjective clinical judgment of a clinician. When searching for the right instrument for screening frailty in community-dwelling older persons, it is also important to keep in mind the dynamic nature of the frailty syndrome. This means that it is easily foreseeable the need of repeatedly screening the target population on a regular basis. Such need poses serious problems in terms of feasibility due to the consequent high demands of resources, budget, and personnel to devote, confirming the implementation of self-reported questionnaires as the most solid solution. Recently, Morley and colleagues proposed the FRAIL questionnaire which has the characteristics for being easily be self-assessed by the older person [15]. Nevertheless, this tool has not the capacity to well differentiate a frail person from one with disability. Such limitation (common to all the existing frailty screening tools) makes this as well as the other instruments inadequate for identifying possible candidates to preventive interventions against disability.

In order to foster the identification of non-disabled older persons at risk of negative health-related outcomes living in the community, we designed the “Frail non-Disabled” (FiND) questionnaire. The novel instrument follows the main multidimensional construct of the widely adopted frailty phenotype [10]. At the same time, it also includes a specific section for excluding the presence of mobility disability (an early stage of the disabling process) [16]–[18]. In the present study, we formally test the agreement of results obtained from the FiND questionnaire with those coming from reference instruments measuring the frailty syndrome and disability in a sample of community-dwelling older persons.

## Methods

### Study sample

A total of 144 individuals randomly drawn from the electoral lists of the general population living in the area of Labastide-Murat (Lot department, France) were invited at undergoing a clinical visit at the Centre Médicale “La Roseraie” (Montfaucon, France). Data are from the 45 subjects accepting to participate (see the “sample size calculation” section).

At the study center, a study physician assessed whether the subject met the eligibility criteria of the study. The inclusion criteria were 1) age of 60 years and older, 2) Mini Mental State Exam (MMSE) [19] score ≥18/30, and 3) absence of any acute disease or injury. Exclusion criteria were 1) failure to provide informed consent, 2) living in nursing home, and 3) systolic blood pressure ≥170 mmHg and/or diastolic blood pressure ≥110 mmHg. In order to not include individuals with acute or subacute conditions preventing the safe conduction of the 400-meter test [20], recent (previous 6 months) overnight hospitalizations for the following conditions (not considered absolute non-inclusion criteria) were investigated: heart attack, stroke, cancer, arthritis, diabetes mellitus, and hip fracture.

Participants were asked to autonomously complete the FiND questionnaire. Then, a blinded assessor objectively measured the frailty and mobility disability status of each participant.

All participants signed an informed consent for participating in the study. The study protocol was approved by the local Institutional Review Board (Comité de Protection des Personnes Sud-Ouest et Outre-Mer, Toulouse).

### The FiND questionnaire

The FiND questionnaire (Table 1) consists of five different questions. Two questions (A and B) are specifically aimed at identifying individuals with mobility disability (an early stage of the disabling process [16], [21]). For the present analyses, the presence of mobility disability was defined as “a lot of difficulties” or “inability” at performing at least one of these two tasks.

**Table 1:** The FiND questionnaire.

Domain	Questions	Answers	Score
*Disability*	**A**. Have you any difficulties at walking 400 meters?	a. No or some difficulties	0
		b. A lot of difficulties or unable	1
	**B**. Have you any difficulties at climbing up a flight of stairs?	a. No or some difficulties	0
		b. A lot of difficulties or unable	1
*Frailty*	**C**. During the last year, have you involuntarily lost more than 4.5 kg?	a. No	0
		b. Yes	1
	**D**. How often in the last week did you feel than everything you did was an effort or that you could not get going?	a. Rarely or sometimes (twice or less/week)	0
		b. Often or almost always (3 or more times per week)	1
	E. Which is your level of physical activity?	a. Regular physical activity (at least 2–4 hours per week)	0
		b. None or mainly sedentary	1

If A+B≥1, the individual is considered as “disabled”.

If A+B = 0 and C+D+E≥1, the individual is considered as “frail”.

If A+B+C+D+E = 0, the individual is considered as “robust”.

Three additional questions (items C–E) were aimed at assessing signs, symptoms, or conditions commonly considered as components of the frailty syndrome [10]: weight loss (item C), exhaustion (item D), and sedentary behavior (item E). In the present analyses, participants presenting one or more frailty criteria in the absence of mobility disability (items A and B) were considered as “frail”. It is noteworthy that the weight loss and exhaustion criteria included in the FiND questionnaire are exactly the same of those originally proposed in the frailty phenotype.

Participants reporting no mobility disability as well as no frailty criterion were considered as robust at the FiND questionnaire.

### Mobility disability

Mobility disability was defined as the incapacity to complete a 400-meter walk test [20]. The dichotomous result of the 400-meter walk test (i.e. ability versus inability to successfully complete the test) has been used in major clinical trials on mobility disability as primary outcome [16], [21]. As mentioned above, mobility disability represents an early stage of the disabling cascade and a proxy for community ambulation. In fact, the 400-meter distance mirrors the minimum distance an older person should be able to cover in order to maintain his/her full independence [16]–[18].

The 400-meter walk test was conducted over a track marked using two cones placed 20 meters apart. Participants were asked to start from a still standing position, walk down the corridor at their usual pace, turn around the cones in a continuous loop, and repeat the course 10 times in order to complete a 400-meter walk. During the test, participants could not use any assistive device. If participants felt the need to stop and rest, they were allowed to do it provided that 1) resumed the walking within 60 seconds, and 2) did not sit down. There were no limits to the number of allowable stops to rest as long as the participant could complete the walk within 15 minutes.

Participants unable to complete the 400-meter walk, taking more than 15 minutes to complete it, and/or sitting during a rest stop were considered to be mobility disabled [22].

### Frailty

The frailty phenotype proposed by Fried and colleagues [10] was used in the present study as reference measure of frailty. The frailty phenotype has shown to be predictive of major health-related negative outcomes in older persons [6]. It was assessed using the five items originally validated in the Cardiovascular Health Study [10], as follows:

- Slow gait speed. Usual gait speed was measured over a 4.57-meter (15-feet) track starting from a standing still position. The slow gait speed criterion was defined as present if the measured gait speed was below the gender- and height-specific cut-points proposed in the original description of the frailty phenotype [10].
- Poor muscle strength. Muscle strength was assessed by using a hand-held dynamometer (model Jamar, Sammons Preston, United Kingdom). Participants were asked to perform the test twice with each hand. The best result was used for the present analyses. The presence of the poor muscle strength criterion was considered as present if below the originally defined thresholds adjusted for gender and body mass index [10].
- Exhaustion. This criterion was considered as present if the participant answered “often” or “most of the time” to either of the two following questions part of the Center for Epidemiological Studies-Depression (CES-D) scale [23]: a) How often in the last week you felt that everything you did was an effort?, and b) How often in the last week you felt that you could not get going?
- Involuntary weight loss. It was defined as unintentional loss of more than 4.5 kg in the past year.
- Sedentary behavior. Participants' physical activity level was measured by means of the Leisure Time Physical Activity questionnaire [24], the modified version of the Minnesota Leisure Time Activity Questionnaire originally used in the Cardiovascular Health study [11], [25]. The criterion was considered as present if the physical activity level fell below the gender-specific thresholds (i.e. <383 kcal/week in men, <270 kcal/week in women) originally proposed by Fried and colleagues [10].

After exclusion of those failing the 400-meter walk test (identified as mobility disabled), the frail, the remaining participants were defined as frail, pre-frail, and robust according to the presence of ≥3, 1-2, and no frailty criteria, respectively [10].

### Statistical analysis

#### Sample size calculation

In a test for agreement between two assessments using the Kappa statistics, a sample of 45 subjects was identifying as achieving 90.4% power (at significance level of 0.05) to detect a true Kappa value of 0.7 in a test of H0: Kappa = 0.30 vs. H1: Kappa <>0.30 [26]. Sample size analyses were conducted considering 3-level categorical variables with frequencies equal to 16% (disabled), 59% (pre-frail and frail), and 25% (robust) as described in literature for the French population [27].

#### Data analysis

Data are presented as percentages, or means ± standard deviations (SD). Cohen's kappa coefficients were calculated to determine the agreement between the FiND questionnaire (and its components) with the reference instruments (i.e. frailty phenotype and mobility disability). For 3×3 tables, quadratic weights (i.e. 1, 0.75, 0) were also applied in the calculation of agreements and kappa coefficients. Sensitivity and specificity of the FiND questionnaire for the identification of non-disabled frail participants were also calculated. SPSS (version 20.0 for Mac, SPSS Inc., Chicago, IL) and Stata (version 12.0SE, StataCorp, College Station, TX) were used for the present analyses.

## Results

Main characteristics of the study sample (n = 45) are presented in Table 2. Mean age of participants was 72.5 (SD 8.2) years, and women were slightly more prevalent than men (62.2% versus 37.8%).

**Table 2:** Characteristics of the study sample (n = 45).

	*Mean ± SD, or n (percentage)*
Age (years)	72.5±8.2
Gender (women)	28 (62.2)
Body Mass Index (kg/m^2^)	26.5±3.8
Mini Mental State Examination	28.2±2.6
Arthritis	21 (46.7)
Cardiovascular disease	7 (15.6)
Diabetes	7 (15.6)
Depression	5 (11.1)
History of cancer	7 (15.6)
Hypertension	14 (31.1)
Osteoporosis	5 (11.1)
Respiratory disease	8 (17.8)
Systolic blood pressure (mmHg)	132.4±17.6
Diastolic blood pressure (mmHg)	73.7±12.4

SD: standard deviation.

Seven (15.6%) participants presented mobility disability as being unable to complete the 400-meter walk test. Participants with mobility disability had a slower gait speed at the 4.57-meter (0.71 [SD 0.49] m/sec) as well as the 400-meter walk (0.25 [SD 0.24] m/sec, taking into account the exact distance they covered, i.e. 154.3 [SD 176.1] m) tests compare to those who were not mobility disabled (1.13 [SD 0.34] m/sec and 1.19 [SD 0.23] m/sec, respectively; all p values <0.01).

According to the criteria proposed by Fried and colleagues [10], 25 (55.6%) participants were defined as pre-frail or frail, and 13 (28.9%) were robust. The prevalence of the frailty criteria (after exclusion of participants with mobility disability) was (in descending order): sedentary behavior (57.9%), exhaustion (13.2%), poor muscle strength (10.5%), slow gait speed (2.6%), and involuntary weight loss (2.6%).

Table 3 presents the comparison of results from the FiND questionnaire and the reference instruments. Overall, it was reported a substantial agreement between the two assessments (84.4%; kappa = 0.748, p<0.001), which also increased when quadratic weights (i.e. 1, 0.75, 0) were applied in the 3×3 table (96.1%, weighted kappa = 0.836, p<0.001). Only 7 (15.6%) participants were incorrectly categorized.

**Table 3:** Comparison of results from the FiND questionnaire with those obtained from the frailty phenotype and 400-meter walk test.

		*Frailty phenotype + 400-meter walk test*		
		*Robust*	*Pre-frail or frail*	*Mobility disabled*
	*Robust*	12 (26.7)	5 (11.1)	0 (-)
***FiND***	*Frail*	1 (2.2)	19 (42.2)	0 (-)
	*Disabled*	0 (-)	1 (2.2)	7 (15.6)

Results are presented as number of participants (percentage of the overall sample).

Unweighted agreement: 84.4%; unweighted Cohen's kappa coefficient: k = 0.748, p<0.001

Quadratic weighted agreement: 96.1%; Quadratic weighted Cohen's kappa coefficient: k = 0.836, p<0.001.

The agreement between results of the FiND disability domain and the 400-meter walk test (dichotomous variable) was excellent (97.8%; kappa = 0.920, p<0.001). For what concerns the FiND frailty domain, the agreements with the reference items of the frailty phenotype were (in descending order): involuntary weight loss (100%; kappa = 1.000, p<0.001), exhaustion (86.7%; kappa = 0.615, p<0.001), and sedentary behavior (77.8%; kappa = 0.537, p<0.001).

Results from analyses aimed at evaluating the capacity of the FiND questionnaire to correctly identify non-disabled frail older persons (that is differentiating the pre-frail and frail subjects from the robust and disabled ones) are presented in Table 4. Again, a substantial agreement of the questionnaire with the reference instruments was found (84.4%; kappa = 0.693, p<0.001). The FiND questionnaire presented a 95% specificity (95%CI 75.1–99.2%) and 76% (95%CI 54.9–90.6%) in the identification of non-disabled frail participants.

**Table 4:** Comparison of results aimed at isolating frail non-disabled participants (thus potential candidates to preventive interventions against disability) with the FiND questionnaire versus the combination frailty phenotype and 400-meter walk test.

		*Frailty phenotype + 400-meter walk test*	
		*Robust or mobility disabled*	*Pre-frail or frail*
***FiND***	*Robust or disabled*	19 (42.2)	6 (13.3)
	*Frail*	1 (2.2)	19 (42.2)

Results are presented as number of participants (percentage of the overall sample).

Agreement: 84.4%; Cohen's kappa coefficient: k = 0.693, p<0.001.

## Discussion

In this study, we formally tested the agreement between a novel self-reported screening tool aimed at identifying non-disabled frail older persons with reference instruments. Aim of the FiND questionnaire is to support the identification of community-dwelling older persons presenting an increased risk profile (i.e. frailty syndrome) in the absence of mobility disability. Main characteristic of the instrument is design allowing the individual's self-assessment. Overall, our study shows a substantial agreement between results of the FiND questionnaire with those obtained from reference assessment tools.

Non-disabled frail older persons are frequently indicated in literature as the ideal target population for preventive interventions against disability in the elderly [1]–[3], [6]. The identification of such individuals (particularly prevalent in our societies) represents a crucial preliminary step in the development of effective prevention against disability and age-related conditions. As occurring for every primary prevention campaign, the screening of a risk factor or early sign of the disease should not be only delegated to general practitioners, but go through a cultural modification in the society. This means that the single individual should be made aware of the modifiable risk condition, its consequences, and the possible available counteractions to take. An increased knowledge about the frailty syndrome and the disabling process in the general population may promote the adoption of healthier lifestyles. Moreover, shifting the screening phase from the general practitioner to the individual him/herself will likely 1) anticipate the identification of possible health problems (thus potentially facilitating the reversion of the risk condition), and 2) reduce the tasks already overcharging the general practitioners' activities.

To our knowledge, the FiND questionnaire is the only assessment tool designed for differentiating frailty from disability. In fact, current frailty instruments provide estimates of the individual's risk profile, but do not inform whether disability is already present. This means that the identification of the non-disabled frail elder could not be conducted without an additional assessment tool specifically measuring the disability status. Such second evaluation is obviously time-consuming and limits the feasibility of the screening, especially when to be applied on a large population. Indeed, the FiND questionnaire fills this gap in the field. In fact, it supports the identification of elders who are experiencing an increased risk of negative events without yet showing signs of mobility disability.

It might be argued that our operationalization might have limited the relevance of gait speed in the definition of frailty, as potentially suggested by the low prevalence of “slow gait speed” after mobility disabled individuals were excluded. It is noteworthy that the choice of focusing the disability assessment only considering the mobility domain is motivated by the hypothesized use of the FiND questionnaire in the framework of preventive actions against disability. By using mobility disability to censor the frailty status, we have confined frailty between robustness and an early stage of the disabling process when the individual is starting to lose his/her capacity to adequately interact with the surrounding environment [16]–[18]. The fact that non-disabled frail older persons present a low prevalence of slow gait speed may be explained by the strong relationship between this parameter and the disability condition. In fact, it is possible that below a certain threshold of gait speed (here operationalized according to the cut-points proposed by Fried and colleagues [10]), it might be particularly unlikely to find non-disabled individuals (especially if the disability definition is centered on the mobility domain as in our study).

The provision of a self-assessment screening tool as the FiND questionnaire may thus support preventive campaigns against disability by indicating to the general population that specific symptoms and signs should not be underestimated and worth to be verified by a clinician. In fact, in a hypothetical scenario, the positive results of the FiND questionnaire should drive the individual at looking for medical advice and verified by the general practitioner (remaining the primary responsible for the individual's health). If the presence of the frailty syndrome will be confirmed, the general practitioner will then consider the need of further diagnostic procedures (e.g. comprehensive geriatric assessment) in order to understand the nature and causes of the frailty syndrome. In particular, the identification of frailty in the absence of mobility disability may support the extension of the comprehensive geriatric assessment technology from its current use (mostly in already disabled individuals) to the novel scenario of disability prevention. Given the large size of the target population (i.e. elders living in the community), it would be otherwise unfeasible delegating to the general practitioner such screening procedures. Furthermore, such hypothetical clinical pathway is common to what already in place for many other preventable diseases. For example, women have been informed about the importance of breast cancer screening and instructed about how to conduct a regular self-assessment. In case of abnormal findings at the self-palpation, the woman seeks for medical advice to plan the eventual diagnostic process.

To date, although the theoretical foundations of the frailty syndrome are quite well described and agreed, no operational definition has been able to attire general consensus. In the present study, we adopted the frailty phenotype [10] as the most commonly used operational definition. Moreover, given its design rendering frailty as a syndrome, this instrument seems particularly suitable for designing preventive strategies against a multidimensional condition as disability. Nevertheless, it should always be considered the difference between the assessment instrument and the measured condition. As the frailty phenotype is not the frailty syndrome *per se* (but its reflection through an *ad hoc* designed instrument), the FiND questionnaire was not designed to propose a “novel” operational definition for the condition of interest. As most of the other existing instruments, it remains a screening tool for a complex and heterogeneous risk condition (i.e. frailty). Therefore, as mentioned above, its positive results should necessarily be followed by a specific diagnostic pathway (i.e. comprehensive geriatric assessment) to understand the underlying biological, clinical, and social foundation of the risk condition.

The present study has several limitations worth to be mentioned. The study population was recruited in a rural area in France. The living environment might modify the frailty profile [28], thus potentially affecting our results. Nevertheless, since the FiND questionnaire was designed for mirroring as much as possible the reference instrument, we would not expect major differences in the agreement when testing other populations. Moreover, it should be considered that the FiND questionnaire is indeed very similar to the original frailty phenotype, having 2 of the 5 original criteria (i.e. involuntary weight loss and exhaustion) exactly replicated. Different results might have been obtained if the FiND questionnaire was tested versus other frailty (e.g. Frailty Index [14]) or disability (e.g. Activities of Daily Living [29]) reference tools. However, the choice of using the frailty phenotype and the 400-meter walk test was motivated by their widespread use and capacity to detect early phases of the disabling process, respectively. Furthermore, the adoption as reference of a frailty instrument rather than another may have secondary importance at this time when a largely agreed operational definition is still lacking. Finally, although based on *ad hoc* sample size analyses, the relatively small number of participants in our study might have affected some of findings. In particular, the evaluation of specific sub-populations in which the agreement of the FiND questionnaire may differ (e.g. subjects with low cognitive function) was not possible. In this context, further studies are needed to confirm and expand our findings.

In conclusion, the proposed screening tool (i.e. FiND questionnaire) may represent an opportunity for diffusing awareness about frailty and disability among community-dwelling older persons and supporting specific preventive campaigns. Moreover, allowing older persons to self-evaluate their health status profile will 1) avoid to delegate the screening of such burdening and highly prevalent conditions to healthcare professionals, and 2) potentially anticipate possible preventive interventions against the disabling process (under the coordination of the general practitioner).
